# The cost of a single concussion in American high school football: a retrospective cohort study

**DOI:** 10.2217/cnc-2020-0012

**Published:** 2020-10-28

**Authors:** Aaron M Yengo-Kahn, Patrick D Kelly, David C Liles, Lydia J McKeithan, Candace J Grisham, Muhammad Saad Khan, Timothy Lee, Andrew W Kuhn, Christopher M Bonfield, Scott L Zuckerman

**Affiliations:** 1 Department of Neurological Surgery, Vanderbilt University Medical Center, Nashville, TN 37232, USA; 2 Vanderbilt Sport Concussion Center, Vanderbilt University Medical Center, Nashville, TN 37232, USA; 3 School of Medicine, Vanderbilt University, Nashville, TN 37232, USA; 4 School of Medicine, Meharry Medical College, Nashville, TN 37208, USA; 5 Department of Orthopedic Surgery, Washington University in St Louis, St Louis, MO 63110, USA

**Keywords:** American football, healthcare costs, post-concussion symptom scale, post-concussion syndrome, sport-related concussion

## Abstract

**Aim:**

The potential financial burden of American football-related concussions (FRC) is unknown. Our objective was to describe the healthcare costs associated with an FRC and determine factors associated with increased costs.

**Methodology/results:**

A retrospective cohort study of concussed high school football players presenting between November 2017 and March 2020 was undertaken; 144 male high school football players were included. Total costs were about $115,000, for an average direct healthcare cost of $800.10/concussion. Visiting the emergency department (β = 502.29, 95% CI: 105.79–898.61; p = 0.01), the initial post-concussion symptom scale score (β = 0.39, 95% CI: 0.11–0.66; p = 0.01) and a post-concussion syndrome diagnosis (β = 670.37, 95% CI: 98.96–1241.79; p = 0.02) were each independently associated with total costs.

**Conclusion:**

A granular understanding of cost-driving factors associated with FRC is the first step in understanding the cost–effectiveness of prevention and treatment methods.

## Lay abstract

The healthcare costs, or the costs incurred by the healthcare system, associated with a single concussion in American high school football are unknown. We reviewed the records of 144 high school football players who received concussion care at our sport concussion center and calculated the direct healthcare costs associated with the appointments, imaging studies and therapies received, when applicable. We found that each concussion results in about $800 of healthcare system spending. A few factors were associated with greater costs, including visiting the emergency department and having symptoms for over 1 month. Furthermore, the more symptoms an athlete had at their first clinic visit, the higher total cost of care. Understanding these costs can potentially help clinicians and researchers determine the best ways to maximize care while minimizing the costs.

A sport-related concussion (SRC) is a mild traumatic brain injury (mTBI) sustained during sports, which is clinically diagnosed and associated with negative standard head imaging, when performed [1]. Nearly one in five male high school athletes sustain a concussion per year, with among the highest rates reported for those who participate in American football [2–4]. American tackle football was associated with $1.35 billion in healthcare costs from 2010 to 2013 [5]. Although this value encompasses more than just concussion, outpatient care was not included, resulting in an underestimation of the total cost of football-related injuries [5–8]. Since concussion is not a hospital-based injury, a gap in SRC cost estimation exists.

While SRC costs will likely vary as treatment evolves [7,9], a fiscal understanding can help determine the willingness of stakeholders to pay for protective equipment, of which the helmet is the most expensive. As SRC occurs by the biomechanical transfer of linear and predominantly rotational accelerations [10], experienced by the head, to the brain, helmets are engineered to reduce this acceleration transfer by distributing impact energy to the various components of the helmet [11]. Depending on the ability to reduce the accelerations experienced by the brain, football helmets can offer different levels of protection [12]. Although these differences may be relatively minor, prices can vary considerably [13], and may be cost-prohibitive in certain communities. An economically sound purchasing decision requires both clinical effectiveness data and the marginal cost of a concussion.

Given the high incidence of football-related concussion (FRC), a granular understanding of the healthcare costs associated with FRC is the first step to ensuring equal prevention and treatment practices for all. The objectives of the present study are to: describe the healthcare costs associated with an FRC, and determine what clinical factors are most associated with increased costs. We hypothesized the development of post-concussion syndrome (PCS) would be particularly associated with higher costs.

## Methods

### Study design & setting

A retrospective cohort study was undertaken between November 2017 and March 2020 for concussed American high school football players. All patients were evaluated and received the majority of their FRC care at a sport concussion center located in the Southeast United States. The study was determined exempt by the Institutional Review Board (IRB #192033) based on minimal risk designation (45 CFR 46.104(d)(4)) and the need for patient consent was waived accordingly following IRB review.

### Patient selection & data collection

**Figure 1.**
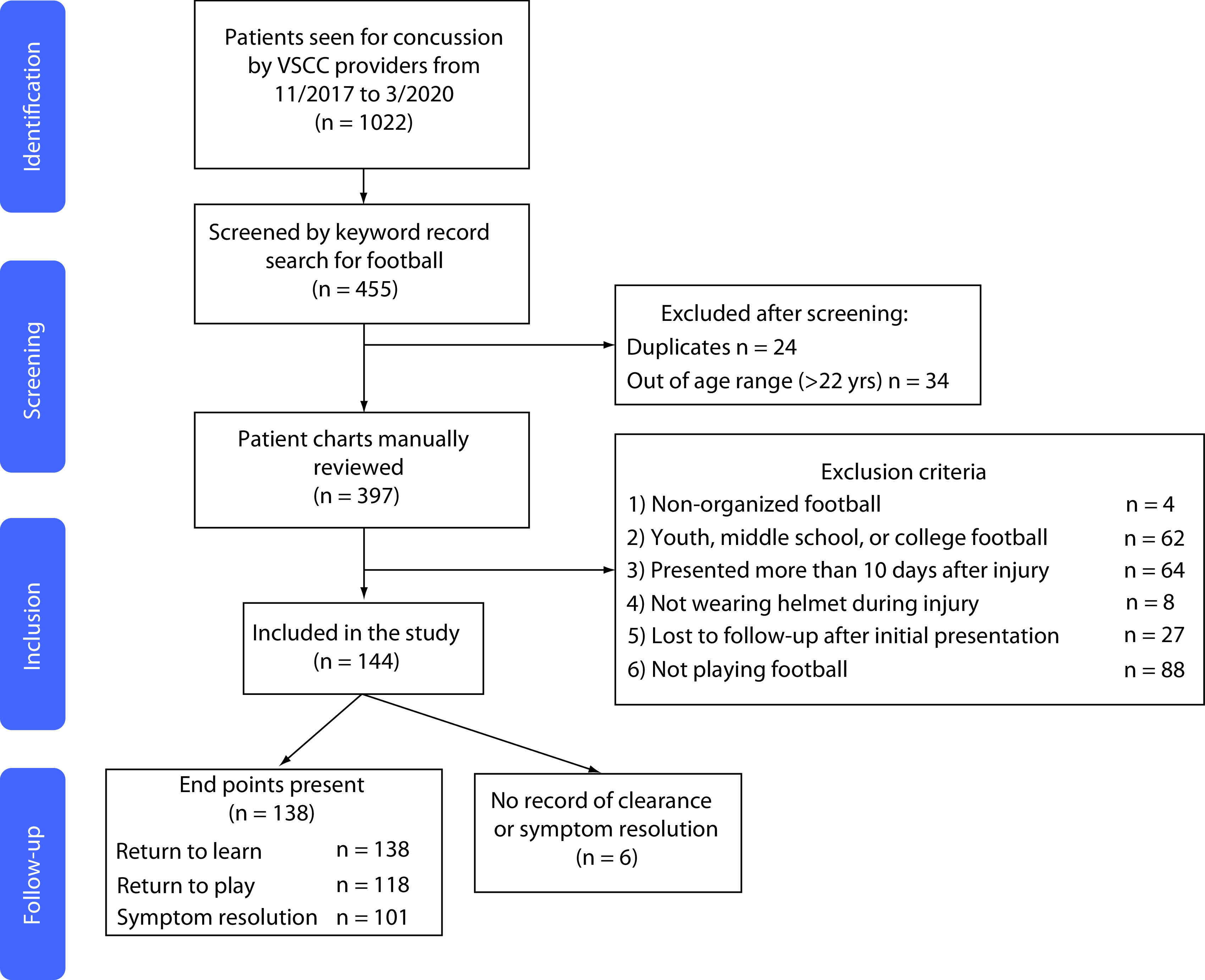
Patient flow diagram. VSCC: Vanderbilt Sports Concussion Center (TN, USA).

All patients seen at the regional sport concussion center during the study period with visit-associated ICD-10 billing codes for concussion (F07.81, S06.0X**) were queried. Patient selection is summarized, and exclusion criteria are detailed in Figure 1. Inclusion criteria were: SRC suffered during high school football game or practice while helmeted, presentation to the concussion clinic within 10 days of injury and this was the first concussion suffered during the study period. Athletes with a history of concussion prior to the study period were eligible for inclusion. These criteria were chosen to limit the inclusion of those who may have received substantial care elsewhere, which would lead to vastly underestimated costs. All variables were extracted by manual chart review using the study institution's electronic health record and stored within a secure database.

### Independent variables

Relevant pre-injury clinical variables, including demographics and medical/family history as well as injury characteristics (loss of consciousness, antero- or retrograde-amnesia) were chosen based on published literature demonstrating importance of these variables in predicting prolonged recovery [14–18]. Initial symptoms and severity upon presentation to sports concussion clinic were documented using the commonly used and validated post-concussion symptom scale (PCSS) [19,20], which contains 22 symptoms rated on a Likert scale from 0 to 6 (least to most severe), yielding a maximum score of 132 [20].

The dates of each concussion-related healthcare system contact were collected. Time (days) from injury to initial evaluation by each type of healthcare provider, as well as the number of visits to each type of provider, were calculated. Emergency department (ED) and urgent care visits were collapsed into a single variable due to an overall low count of urgent care visits and the two provide a similar service involving the initial assessment and diagnosis of SRC. Imaging was identified by radiology reports and imaging studies were verified to be SRC-related by reviewing temporally associated provider notes. The presence of outside facility imaging studies, urgent care or ED use was determined by close review of the initial concussion clinic consultation note, as these variables are systematically documented as part of our institution's initial clinic evaluation.

### Concussion episode end points

A concussion episode was defined as time from initial injury to discharge from the clinic, which typically occurred when the athlete was cleared for return to play or became asymptomatic as athletes typically return to school prior to discharge from clinic. Four clinical outcomes, including three time-to-recovery variables, were chosen *a-priori* as end points. Return-to-learn: days from injury to return to school (part or full time); return-to-play: days from injury to full clearance for return to practice and/or games; symptom resolution: days from injury to when PCSS was 0 or the treating clinician stated the athlete had ‘recovered’ from his concussion; and PCS: whether or not the athlete developed PCS, defined as >1 symptom continuing over 1 month (28 days) post-injury [21]. If an athlete was released from the clinic into the care of their athletic trainer prior to reaching an end point, it was inferred the athlete did not develop PCS.

### Primary outcome

The direct cost of the concussion episode was the primary outcome. Each patient's cost data was derived by matching visit and imaging dates with study institution inflation-adjusted cost data. Notably, costs were used instead of charges, which can fluctuate greatly by region/hospital [22,23]. Costs were inflation-adjusted to December 2017 dollars using the Bureau of Labor Statistics Producer Price Index for Outpatient Hospital Care at General Medical or Surgical Hospitals [24]. Costs were set at December 2017 dollars rather than April 2020 levels due to SARS-CoV-2 pandemic-related disruption of the inflation cost curve [25]. Cost data were presented in two forms similar to prior studies [8]. To obtain the primary outcome of direct cost per SRC episode, total costs were divided by total SRCs. Secondarily, the cost per resource utilization represents the total cost of a given healthcare resource divided by the number of patients who utilized it (e.g., the average costs associated with that resource).

### Statistical analysis

Descriptive statistics were performed, with continuous variables represented as median with IQR and categorical variables as N (%). Sample size was determined by the number of available records, and thus no sample size or power calculation was performed. Missing data were imputed using mean value imputation. Three patients – two with non-contrasted head CTs (CTH), one with a cervical spine (c-spine) MRI – underwent in-system imaging related to their concussions, for which cost data were missing, and these costs were imputed by averaging inflation-adjusted costs for CTH and c-spine MRI collected from other patients. For patients receiving outside imaging and/or outside ED care, the costs were imputed utilizing the mean in-system cost of these services. Wilcoxon rank-sum tests were used to compare the initial symptom burden and total costs incurred by those who did and did not develop PCS given non-normally distributed data.

A multivariable linear regression model identified clinical factors associated with the total cost of concussion care. In-system ED visits were included as a covariate to mitigate the confounding influence of this variable. Based on the anticipated relationship between PCSS and cost, a nonlinear quadratic term for this variable (PCSS^2^) was included in the regression model.

The threshold for statistical significance was set *a priori* as p < 0.05. No sensitivity analyses were performed. All statistical analyses were performed in Stata/IC version 16.1. (StataCorp LLC, TX, USA).

## Results

### Patient demographics, injury characteristics & outcomes

Demographics, clinical characteristics and outcomes of the 144 concussed high school football players are presented in Table 1. At least one of the three, time-to-recovery, end points was able to be ascertained for 138 (95.8%) patients. Six patients (4.2%), were instructed to only return to the clinic as needed after the initial visit and therefore no further outcome data were obtained. PCS status was able to be determined or inferred based on management for all patients.

**Table 1.** Biopsychosocial characteristics, clinical attributes and outcomes of cohort.

Factor	n^†^ (total n = 144)
Male	144 (100%)
Age at concussion [median (IQR)]	16 (15–17)
Race White/Caucasian Black/African–American Other Unknown	79 (54.9%) 43 (29.9%) 5 (3.5%) 17 (11.8%)
Personal medical history Attention deficit hyperactivity disorder Migraine Psychiatric Learning disabilities	21 (14.6%) 14 (9.7%) 8 (5.6%) 10 (6.8%)
Family medical history Migraine Psychiatric	32 (22.2%) 10 (6.9%)
Number of prior concussions [median (IQR)]	0 (0–1)
Loss of consciousness	22 (15.3%)
Amnesia	31 (21.5%)
Initial PCSS score (median, IQR)	11 (1, 27.5)
**Outcomes** (days) Return to learn [median (IQR)] Return to play [median (IQR)] Symptom resolution time [median (IQR)] Post-concussion syndrome, n (%)	4 (3–9) [n = 138] 12 (6–21) [n = 118] 10 (5–16) [n = 101] 14 (9.7%) [n = 144]

† Unless otherwise noted.

IQR: Interquartile range.

### Costs & healthcare system utilization

Complete healthcare system utilization and costs per concussion and costs per resource utilization are presented in Table 2. The total cost of concussion care in the study population was $115,214.40, resulting in an average cost of $800.10 per concussion. The ED or an urgent care clinic was utilized by over a quarter of the cohort (27.8%) at $687.86 per use, and 80.5% of these visits occurred within 1 day of the initial injury. Imaging was performed for 12.9%, 1 patient received a repeat CTH (0.7%). Both occupational therapy and psychiatry had substantial associated costs, in excess of $1000 per athlete referral, related to multiple visits (medians 11.5 and 3.5, respectively) despite only being utilized by 1.4% of the cohort.

**Table 2.** Care utilization and associated costs for a single concussion episode.

Care factor	n^†^	Cost per concussion episode^‡^	Cost per utilization^‡^
***Average total cost of concussion***	144 (100%)	800.10 ± 1072.51	–
***Number of total healthcare visits*** [median (IQR)]	2 (1–3)	–	–
ED or urgent care evaluation In-system Outside facility Days from injury to visit [median (IQR)]	**40 (27.8%)** 21 19 0 (0–1)	190.74 ± 504.81	687.86 ± 993.65
Imaging performed CT head MRI brain C-spine XR C-Spine MRI	**19 (12.9%)** 17 (11.6%) 0 (0.0%) 4 (2.7%) 2 (1.4%)	18.99 ± 52.77 0.00 2.08 ± 11.51 7.73 ± 53.23	148.66 ± 90.25 0.00 60.00 ± 28.16 371.26 ±13.50
Sports medicine/concussion clinic Overall utilization Number of visits [median (IQR)] Days from injury to clinic [median (IQR)]	140 (97.2%) 2 (1–2) 3.5 (1–6)	453.63 ± 356.19	466.59 ± 352.74
Neurology Overall utilization Number of visits [median (IQR)] Days from injury to clinic [median (IQR)]	7 (4.9%) 1 (1–1) 26 (22–105)	23.53 ± 134.01	423.53 ± 416.26
Neuropsychology Overall utilization Number of visits [median (IQR)] Days from injury to clinic [median (IQR)]	7 (4.9%) 1 (1–3) 7 (5–15.5)	29.42 ± 164.95	706.20 ± 452.37
Psychiatry Overall utilization Number of visits [median (IQR)] Days from injury to clinic [median (IQR)]	2 (1.4%) 3.5 (2–5) 158 (112–204)	17.96 ± 188.46	1,293.31 ± 1,334.13
Occupational therapy Overall utilization Number of visits [median (IQR)] Days from injury to clinic [median (IQR)]	2 (1.4%) 11.5 (8–15) 28 (20–48)	39.75 ± 304.39	1,908.00 ± 1,130.37
Physical therapy Overall utilization Number of visits [median (IQR)] Days from injury to clinic [median (IQR)]	4 (2.8%) 2 (1.5–3) 32 (19–48)	10.03 ± 88.68	481.41 ± 471.10

† Unless otherwise noted.

‡ Presented as mean ± standard deviation.

C-spine: Cervical spine; CT: Computed tomography; ED: Emergency department; IQR: Interquartile range; MRI: Magnetic resonance imaging; XR: x-ray.

### Predictors of cost

**Figure 2.**
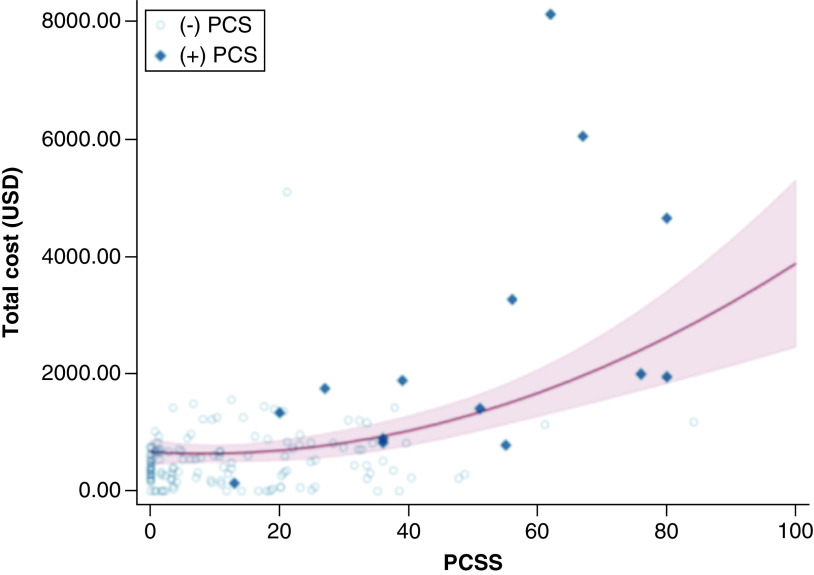
Scatterplot demonstrating the relationship between initial symptom burden and total cost of the concussion episode. The trend line demonstrates the marginal effect of the linear and quadratic post-concussion symptom scale (PCSS) terms on the total cost of concussion care. Individuals who went on to develop post-concussion syndrome (PCS) are designated with diamond markers and tend to cluster toward high initial symptom burden (PCSS) and higher costs.

Multivariable linear regression revealed that presenting to any ED or urgent care was independently associated with total cost (β = 502.20, 95% CI: 105.79–898.61; p = 0.01). Additionally, there was a statistically significant association between the squared PCSS and total cost (β = 0.39, 95% CI: 0.11–0.66; p = 0.01), indicating a quadratic relationship (Figure 2). Accordingly, being diagnosed with PCS was also independently associated with higher cost (β = 670.37, 95% CI: 98.96–1241.79; p = 0.02) (Table 3). Athletes who developed PCS had higher initial symptom burdens (Wilcoxon rank-sum; p < 0.01) and incurred a greater average cost of $2579.70 compared with $608.45 for those without PCS (Wilcoxon rank-sum; p < 0.001).

**Table 3.** Multivariate linear regression of factors related to concussion cost.

Factor	β	p-value	95% CI LCL	95% CI UCL
Age	35.25	0.47	-60.15	130.65
Prior concussion	-163.26	0.25	-443.20	116.69
Migraine history	9.96	0.96	-438.15	458.08
FHx of migraine	262.60	0.15	-93.21	618.40
FHx psychiatric illness	317.24	0.26	-235.64	870.12
Amnesia	-110.12	0.52	-445.72	225.48
Loss of consciousness	-8.89	0.96	-382.05	364.28
Initial PCSS	-6.38	0.50	-24.84	12.09
**Initial PCSS^2^**	**0.39**	**0.01^†^**	**0.11**	**0.66**
**ED or urgent care**	**502.20**	**0.01^†^**	**105.79**	**898.61**
In-system ED visit	499.73	0.07	-44.32	1043.79
**PCS**	**670.37**	**0.02^†^**	**98.96**	**1241.79**
Constant	-190.80	0.81	-1781.64	1400.03

† Significant at p < 0.05.

LCL: Lower confidence limit; UCL: Upper confidence limit; ED: Emergency department; FHx: Family history; PCS: Post-concussion syndrome; PCSS: Post-concussion symptom scale; PCSS^2^: Quadratic term of initial PCSS.

## Discussion

We sought to determine the average direct cost to the healthcare system of an FRC among US high school athletes. Over $115,000 was spent across 144 football SRCs for an average cost of $800 per concussion. Our initial hypothesis was supported, as a diagnosis of PCS was independently associated with higher costs and athletes. Additionally, we found that both visiting the ED and a higher initial symptom burden were independently associated with increased costs. These results represent one initial attempt to quantify the direct costs of SRC, and describe the relationship between symptom burden, prolonged recovery and direct healthcare costs for male, high school athletes who suffer a concussion playing football.

### Costs in context

Many investigations have quantified costs associated with mTBI [6,8,26,27]. However, most studies include only inpatient and ED costs, which make them not applicable to SRC since very few, if any, patients are admitted to the hospital [5,26,28]. In pediatric patients with general mTBI, Taylor *et al.* [8] found a slightly less expensive cost of $608 than our $800 per injury, after inflation adjustment [24]. This difference may be related to our football specific, all-male cohort with an older age range, as increasing age is associated with increased costs in the 1 year post-mTBI [6]. Alternatively, this discrepancy may be related to continuous exponential growth of healthcare spending [29]. Furthermore, the Taylor *et al.* [8] study reported costs to the payer (i.e., reimbursement), which is more susceptible to outside forces (negotiating power of hospital vs insurer) than provider costs, and may represent declining reimbursement rather than more efficient care [4,12,30].

### Cost drivers & considerations

Emergency room evaluation was independently associated with a higher cost per concussion episode. The indication for an ED visit was not standardized nor documented, but red flags symptoms (intractable nausea, altered mental status) may have been present, warranting the visit. Improving the efficiency of ED utilization represents a potential method to reduce costs. For example, limiting CTH to evidence-based indications [31], improving ED discharge instructions to emphasize the low yield of standard imaging [32] and early specialty referrals may improve cost. Early specialty concussion clinic referral has been associated with faster recovery time [33], therefore prompt concussion clinic referral, in lieu of an ED visit, may reduce costs without sacrificing care.

Initial symptom burden was strongly associated with costs. Though initial symptom score has been associated with protracted recovery [17,30], to our knowledge this is the first instance where highly symptomatic (PCSS >20) athletes and those with PCS were associated with increased costs of concussion care. Interventions to prevent PCS may reduce overall costs. Recent trials have demonstrated that early subthreshold aerobic exercise was associated with a 10% absolute risk reduction for delayed recovery (i.e. PCS) [9]. Quantifying the cost of concussions is the first step in evaluating the cost–effectiveness of other treatment interventions.

### Cost–effectiveness of football helmets

Over the last decade, helmet research has shifted toward concussion prevention and risk reduction [34–36]. Despite robust engineering analyses [35,37,38], little attention has been directed toward the costs to achieve these reductions. Like any healthcare intervention, protective equipment should be subject to cost–effectiveness analysis and transparency regarding the cost per incremental improvement.

Consider the hypothetical $525 upgrade from the Riddell Speedflex (MSRP $425 [39]) to the VICIS Zero 1 (MSRP $950 [40]). Early reports suggest that the absolute risk reduction by making this change was 3.3% [41], and therefore 30 helmet upgrades are needed to prevent one concussion. Thus, societal costs to prevent one concussion equates to $15,909. However, the direct healthcare cost of an FRC appears to be only $800. This equation oversimplifies a complex relationship that includes indirect costs of missed school/work and long-term cognitive outcomes. Although simple, this example illustrates the economic disparity between prevention and treatment. Scientific progress cannot move forward without economic awareness and, at present, $15,000 spent to upgrade helmets is likely not the best use of financial resources. Funding may be better spent on hiring full-time athletic trainers focused on improving post-concussion care and mitigating PCS risk [42,43]. Furthermore, strategies aimed at preventing concussion by limited contact practices or changing gameplay rules (i.e., advancing the kickoff position) represent cost-free interventions that may considerably reduce concussion burdens [44,45].

### Strengths & limitations

The primary strength of this study is providing an initial attempt to provide a granular accounting of the direct costs associated with FRC. Compared with other ‘cost’ studies, a major strength of this study lies in the use of hospital cost data rather than billing charges or reimbursement. Charges can greatly overestimate direct costs and cannot be compared across multiple hospital systems while reimbursement can similarly change due to insurer, hospital market share and an individual's ability to pay [22,23,46]. Therefore, true hospital cost is likely the most objective method to gauge total costs related to concussion. The ability to precisely review line-item costs on a patient-by-patient basis maximizes the granularity of our cost estimates compared with studies utilizing insurance claims data, for instance. Furthermore we were able to record clinical outcomes for the majority of athletes to demonstrate their recovery trajectories were similar to those previously published [33,47,48].

Despite these strengths, there are important limitations to this study in addition to regional care differences that may limit generalizability. First, direct costs of concussion care reflect those incurred by the health system and these results do not account for costs attributable to FRC that occur outside our health system. Imputed imaging and ED costs mitigate this limitation and inclusion criteria were deliberately set to include athletes who would have received the vast majority of their care within our hospital system. Second, we decided to focus on direct costs rather than providing estimates of indirect costs. Specifically, we did not include the costs of health-related quality-of-life (HRQOL) detriments due inconsistent effect of sports-related concussion on HRQOL among studies ranging from no difference [49] to substantial declines that resolve at the time of symptom resolution [50]. Additionally, a conservative estimate of indirect costs suggested these represent 4% ($32 = 36 x 20 x [16/365]) or less of total costs, calculated using a substantial decline of about 20 QOL points [50] lasting until symptom resolution at 16 days (75th percentile) and an assumption of $36 per year per HRQOL point [51]. Therefore, given our current poor understanding of the costs associated with transient declines in HRQOL, we decided to focus on direct costs exclusively.

Similarly, our study reflects the relatively acute or initial costs associated with FRC. There is emerging evidence of psychological maladies associated with FRC that may lead to higher longitudinal healthcare costs [52,53]. Yet, as young athletes accumulate life experience following their concussions, drawing a link between a remote FRC and the new development of psychological issues requiring treatment becomes increasingly difficult. Future longitudinal studies may better elucidate what constitutes the delayed costs associated with FRC.

Finally, we focused on a single sport for this initial investigation given the high incidence of FRC and the expenses associated with injury prevention specific to this sport. Future investigations can expand to understand differential healthcare utilization across sports and decision-tree analyses may determine the cost-effectiveness of rehabilitation-based versus equipment-based interventions.

## Conclusion

The average cost of a concussion suffered playing high school American Football in the Southeastern USA is approximately $800. Costs are independently associated with the initial symptom burden, ED utilization and/or development of PCS. A granular understanding of the costs and cost-driving factors associated with concussions is a critical first step to study the cost–effectiveness of prevention and treatment methods. We encourage further investigations of the cost–effectiveness of SRC-related interventions in order to make sound economic and scientifically based decisions.

## Future perspective

As the prevention and treatment of SRC continues to advance technologically and scientifically, the potential costs to athletes, teams, society and the healthcare system have the potential to expand indefinitely. As these costs mount, attention must be placed on the costs per unit benefit. Stakeholders will demand that these rising costs are matched with benefits in the form of markedly reduced recovery time, reduced risk of any long-term effects of SRC or improved primary protection from SRC. Simple advances that achieve meaningful improvements with near negligible costs will be preferred to significant technological advancements accompanied by disproportionate costs that yield only minor improvements over existing products, methods and treatment standards.

**Summary points**

**Background**

- American high school football-related concussions (FRC) are exceedingly common.
- The healthcare costs of these injuries and what factors drive greater costs have not been previously described.

**Participants**

- A total of 144 American high school football players who suffered an FRC were included.
- All players received care through a regional, multidisciplinary concussion clinic.

**Direct healthcare costs**

- The direct healthcare cost per concussion was about $800.
- These costs reflect the direct cost of care to the healthcare system, providing an objective cost measure.
- These costs are objective, reproducible and more generalizable to other health systems, unlike costs/charges as assessed by the insurance company or experienced by patients as these others costs/charges are highly variable across carriers and patients.

**Cost drivers**

- Visiting the emergency department, higher initial symptom burden and developing post-concussion syndrome each independently drove higher healthcare costs.

**Conclusion**

- This provides a calculation of the direct healthcare costs of a sport-related concussion (SRC) in any sport as well as demonstrates the association between symptom burden and costs for the first time.
- Understanding the costs associated with SRC provides the groundwork for demonstrating cost-effectiveness of interventions – both preventative and treatment – in the future.
